# Serum Vitamin D Level and the Risk of Urinary Tract Infection in Children: A Systematic Review and Meta-Analysis

**DOI:** 10.3389/fpubh.2021.637529

**Published:** 2021-03-19

**Authors:** Xiaoyan Li, Qing Yu, Feng Qin, Biyu Zhang, Yanming Lu

**Affiliations:** Department of Pediatrics, Renji Hospital, Shanghai Jiaotong University School of Medicine, Shanghai, China

**Keywords:** urinary tract infection, vitamin D, children, meta-analysis, UTI

## Abstract

This systematic review and meta-analysis aimed to evaluate the association between serum vitamin D concentration and the risk of urinary tract infection (UTI) in children. Human studies reported the serum vitamin D level in children with UTI and healthy controls were collected from PubMed, Scopus, Embase, and Cochrane databases. The strictly standardized mean difference (SSMD) and 95% confidence interval (CI) were calculated to evaluate the relationship between serum vitamin D levels and risk of UTI. The results of analysis showed that serum vitamin D levels in children with UTI were significantly lower than healthy control children (SSMD: 0.891, 95% CI: 0.707–1.075, *p* < 0.000; SSMD: 0.797, 95% CI: 0.500–1.094, *p* < 0.000, respectively). It can be concluded that there is a significant negative relationship between serum vitamin D level and risk of UTI in children.

## Introduction

Urinary Tract Infection (UTI) is one of the most common bacterial infections in childhood. It is categorized based on the level of involvement of urinary system to cystitis and pyelonephritis (1). The incidence of UTI varies from 1.8 to 7.5% in childhood (2). UTI is more common in boys under 3 months than girls. However, girls are more likely to develop UTI after this age (3). Understanding the pathogenesis and risk factors of UTI in children is crucial because of its associated serious complications.

Bacterial virulence, immunodeficiency, nutritional deprivations and anatomic or functional abnormalities make children vulnerable to UTI (4). *Escherichia coli* (*E. coli*) are the most important pathogen in children with UTI. Other bacteria including *Klebsiella, Proteus, Enterobacter*, and *Pseudomonas* as well as fungal pathogens are also involved in the pathogenesis of UTI (5). *E. coli* is known as the predominant pathogen for UTI due to their attachment to the endothelial wall of the urinary tract and found in 90% of girls and in 80% of boys at the primary childhood UTI (6). UTI actually occurs when bacteria enter the urinary tract through the urethra, intestine, blood or lymph and begin to proliferate. Antibiotics are the first and most important treatment for UTI, and the severity of the infection and the type of bacteria determine the type of antibiotic used. However, antibiotic therapy may lead to antibiotic resistance, a serious threat to global public health (7).

The use of micronutrients, natural products and vitamins are becoming more popular in different diseases in children (4, 8). The use of vitamin D as a supplement in prevention and treatment of UTI and its complications has been reported (9). Moreover, the association between vitamin D and several infectious diseases has been studied for a long time (10). On the other hand, there is increasing evidence that vitamin D deficiency plays an important role in susceptibility to UTI and administration of vitamin D can prevent it. It was reported that supplementation with vitamin D might prevent UTI (9). In addition to, several studies demonstrated that the low level of 25(OH)D may be a risk factor for UTI (11–15). Beside the growing evidence on the role of vitamin D supplementation in prevention and treatment of UTI in children, no study has systemically evaluated the association between serum vitamin D level and risk of UTI in children. Therefore, the current meta-analysis is planned to determine whether the serum level of vitamin D is associated with the risk of UTI in children.

## Materials and Methods

### Data Sources and Search Strategy

Human studies reported the serum vitamin D levels in children with urinary tract infection and healthy controls were searched databases of PubMed, Scopus, Embase, and Cochrane. The terms of “vitamin D,” “25-Hydroxyvitamin D,” “vitamin deficiency,” “urinary tract infection,” “child,” “children,” “pediatric” in subject, abstract and keywords were searched in these databases. Studies published from the inception of the databases to 31th December 2020 have been included in the review. The search for literature was limited to articles in English. The articles were screened by checking the title and the abstract and those which were related to our study were selected for further assessments. Then, the full text articles were reviewed for eligibility. The articles which were not available online in full text were requested from the corresponding author through email.

### Eligibility Criteria

Only human studies reported the serum vitamin D levels in children with urinary tract infection and healthy controls were included. Animal studies, reviews, letters, commentaries, clinical cohorts without appropriate controls and case studies were excluded. In the case of duplicated reports, only the paper which was in more details was enrolled in the study.

### Quality Assessment

The methodological quality of the included studies in this meta-analysis was evaluated by a checklist from JBI (Table 1). These criteria include clearly definition of inclusion criteria, study subjects and the setting described in detail, valid and reliable measurement method, use standard and objective criteria, identifying confounding factors, using strategies to counter confusing factors, measuring outcomes in a valid and reliable way and using appropriate statistical analysis. Studies that answered yes to at least four questions were used to extract the data.

**Table 1:** Characteristics of included studies.

**Study**	**Country**	**Year**	**Study design**	**Mean ± SD age all**	**N disease**	**N control**	**Mean ± SD vitamin D in disease (nmol/l)**	**Mean ± SD vitamin D in control (nmol/l)**
Georgieva et al. (16)	Sweden	2019	Cross-sectional study	Under 3 years	76	44	80.8 ± 21.2	101.1 ± 33
Noorbakhsh et al. (17)	Iran	2019	Prospective cohort study	2.17 y	25	40	114.25 ± 52.625	114.75 ± 62
Mahyar et al. (11)	Iran	2018	Case-control study	Patients: 53.2 ± 35.6months Control: 36.1 ± 60.2 months	70	70	51 ± 21.5	42.25 ± 18.5
Shalaby et al. (15)	Egypt	2018	Prospective case-control study	2 months−6 years	50	50	10.5 ± 2.7	27.9 ± 5.6
Övünç Hacihamdioglu et al. (18)	Turkey	2016	Cross-sectional prospective study	Patients:6.8 ± 3.6 y Control:6.3 ± 2.8 y	36	38	10.5 ± 2.7	59.25 ± 27.5
Tekin et al. (12)	Turkey	2014	Controlled prospective study	Patients: 2.57 ± 2.56 y Control: 2.10 ± 1.37 y	82	64	29.25 ± 8.25	69 ± 11.75

### Data Extraction

Data were extracted independently by two reviewers based on predefined protocol (including: the first author and published year, country, study design, age of participants, sample size in control and in patients, study population and serum level of vitamin D in control and in patients). All the obtained information were read and evaluated by two independent reviewers. The unit of vitamin D was transformed to nmol/L through SI units Conversion Calculator (http://unitslab.com/node/84) in case of report in other units.

### Statistical Analysis

The statistical association between serum level of vitamin D and risk of urinary tract infection by analyzing of the mean ± standard deviation (SD) of serum vitamin D level, sample size and age of samples in control and patient groups was evaluated. One of the modification of data that was made in this study convert different units (such as ng/mL) of vitamin D to nmol/L and convert the standard error of the mean (SEM) to standard deviation (SD). Heterogeneity tests including Cochran χ^2^ and *I*^2^ statistics were performed to minimize variations in vitamin D levels among studies. In the absence of statistical heterogeneity between studies the fixed effects model was used, otherwise the random effects model was employed. Fixed and random effects were represented as strictly standardized mean difference (SSMD) with 95% CI. SSMD with 95% CI were employed to evaluate the effects of vitamin D status on the risk of urinary tract infection in different ages of children. Sensitivity analysis was performed by removing each study from the pooled analysis. Subgroup analysis was also performed in our study on the basis of the different age groups. The bias in included studies was evaluated by Egger's test and Begg's test. All statistical analyses were performed using CMA version 2. *P*-value < 0.05 was considered statistically significant.

## Results

### Description of Search

**Figure 1:**
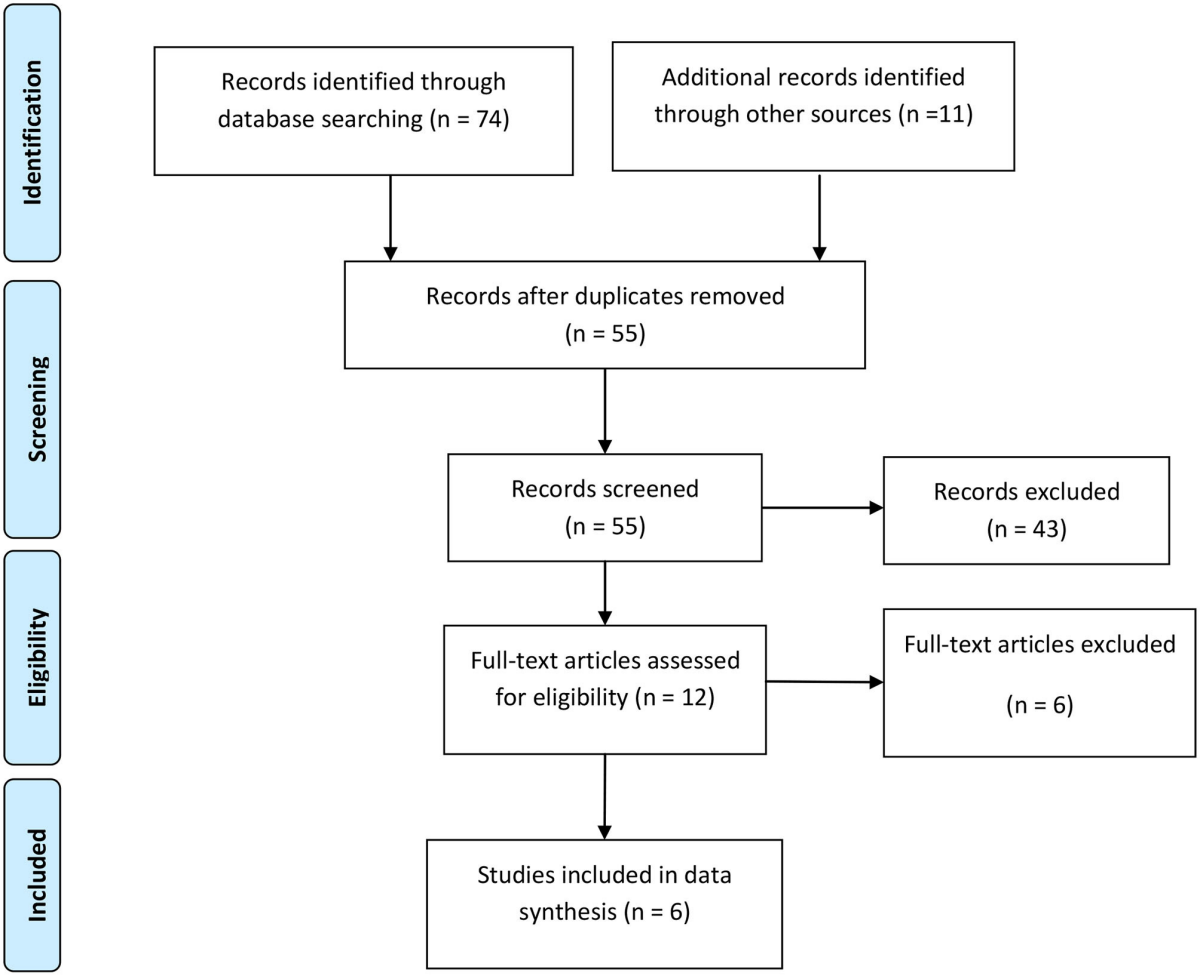
PRISMA flow diagram for included studies.

After complete search of the predefined databases, 85 titles were retrieved. Omitting duplicate topics, 55 titles were evaluated in terms of topic and abstract. The full texts of 12 articles were examined and 6 articles entered the systematic review. Among 49 excluded studies in the stages of topic, abstract and full text evaluation 24 studies were excluded because of lacking control group, 21 studies excluded for not matching patient population, and 4 studies were case reports and review. Six studies including 339 children with urinary tract infection cases and 306 healthy controls were included in the meta-analysis. The flowchart of the included studies is presented in Figure 1.

### Characteristics of Included Studies

The characteristics and quality assessment of the six included studies are presented in Table 1. This Table contains one case-control studies (11), one cross-sectional prospective study (18), one controlled prospective study (12), one prospective case-control study (15), one cross-sectional study (16) and one prospective cohort study (17). Two studies were conducted in Turkey (12, 18), two in Iran (11, 17), one in Egypt (15), and one in Sweden (16). The assessment of the methodological quality of the included studies is presented in Table 2.

**Table 2:** JBI critical appraisal checklist applied for included studies.

**References**	**Q1**	**Q2**	**Q3**	**Q4**	**Q5**	**Q6**	**Q7**	**Q8**	**Overall quality**
Georgieva et al. (16)	Yes	Yes	Yes	Yes	No	No	Yes	Yes	4/8
Noorbakhsh et al. (17)	Yes	Yes	Yes	Yes	No	No	Yes	Yes	5/8
Mahyar et al. (11)	Yes	Yes	Yes	Yes	No	No	Yes	Yes	4/8
Shalaby et al. (15)	Yes	Yes	Yes	Yes	No	No	Yes	Yes	4/8
Övünç Hacihamdio glu et al. (18)	Yes	Yes	Yes	Yes	No	No	Yes	Yes	5/8
Tekin et al. (12)	Yes	Yes	Yes	Yes	No	Yes	Yes	Yes	6/8
Q1. Were the criteria for inclusion in the sample clearly defined?									
Q2. Were the study subjects and the setting described in detail??									
Q3. Was exposure measured in a valid and reliable way?									
Q4. Were objective, standard criteria used for measurement of the condition?									
Q5. Were confounding factors identified?									
Q6. Were strategies to deal with confounding factors stated?									
Q7. Were the outcomes measured in a valid and reliable way?									
Q8. Was appropriate statistical analysis used?									

### Serum Vitamin D Levels in Children With and Without UTI

**Figure 2:**
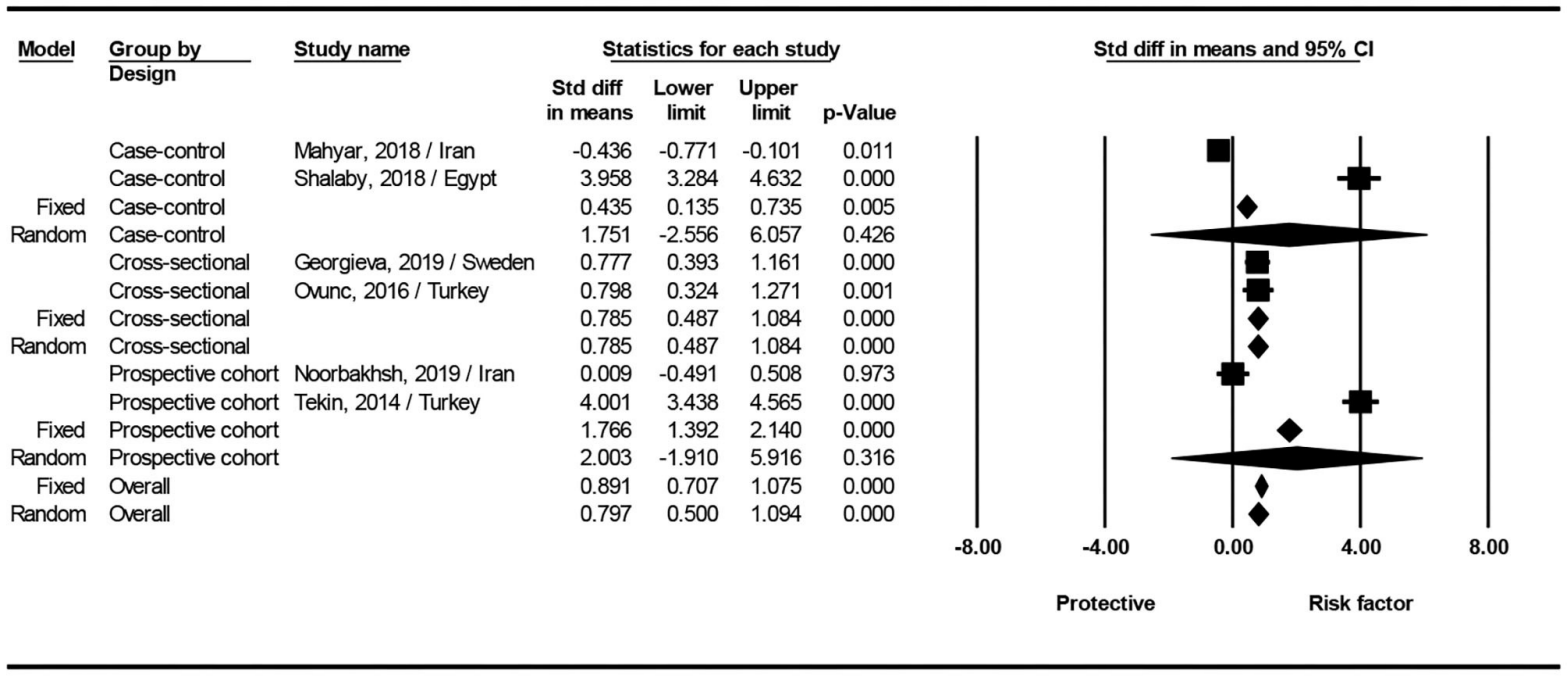
Forest plots showing the association between vitamin D level with the risk of urinary tract infection in children.

Four of the six included studies showed that serum levels of vitamin D were significantly lower in children with urinary tract infection than that in controls (14–16, 18). One study by Noorbakhsh et al. (17) showed no significant differences between the patient and control groups. Interestingly, one study found that children with urinary tract infections had higher serum levels of vitamin D than healthy children (20.4 ± 8.6 ng/mL vs. 16.9 ± 7.4 ng/mL) (11). Interestingly, data from Figure 2 show that the values obtained for vitamin D in the two Mahyar et al. (11) and Noorbakhsh et al. (17) studies have a protective role against UTI. However, other data show that the values of vitamin D in other studies act as a risk factor for UTI (12, 14, 15, 18), in other words, low levels of vitamin D are known as a risk factor for UTI. Sum up, analysis of the all groups showed that serum levels of vitamin D can be a risk factor for UTI (SMD: 0.797; 95% CI 0.500–1.094, *p* < 0.001; Q-value = 269.2, df = 5, *I*^2^ = 98.1, *p* < 0.001).

### Subgroup Analysis

The result of subgroup analysis based on study design show that for case-control (SMD: 1.751; 95% CI; −2.556 to 6.057, *p* = 0.426; Q-value= 130.8, df = 1, *I*^2^ = 99.2, *p* < 0.001), and prospective cohort studies (SMD: 2.003; 95% CI; −1.910 to 5.916, *p* = 0.316; Q-value = 107.9, df = 1, *I*^2^ = 99.0, *p* < 0.001), no statistical difference found between two groups. In cross-sectional studies significantly serum levels of vitamin D can be a risk factor for UTI (SMD: 0.785; 95% CI 0.487–1.084, *p* < 0.001; Q-value = 0, df = 2, *I*^2^ = 0.0, *p* = 0.947) (Figure 2).

### Publication Bias

**Figure 3:**
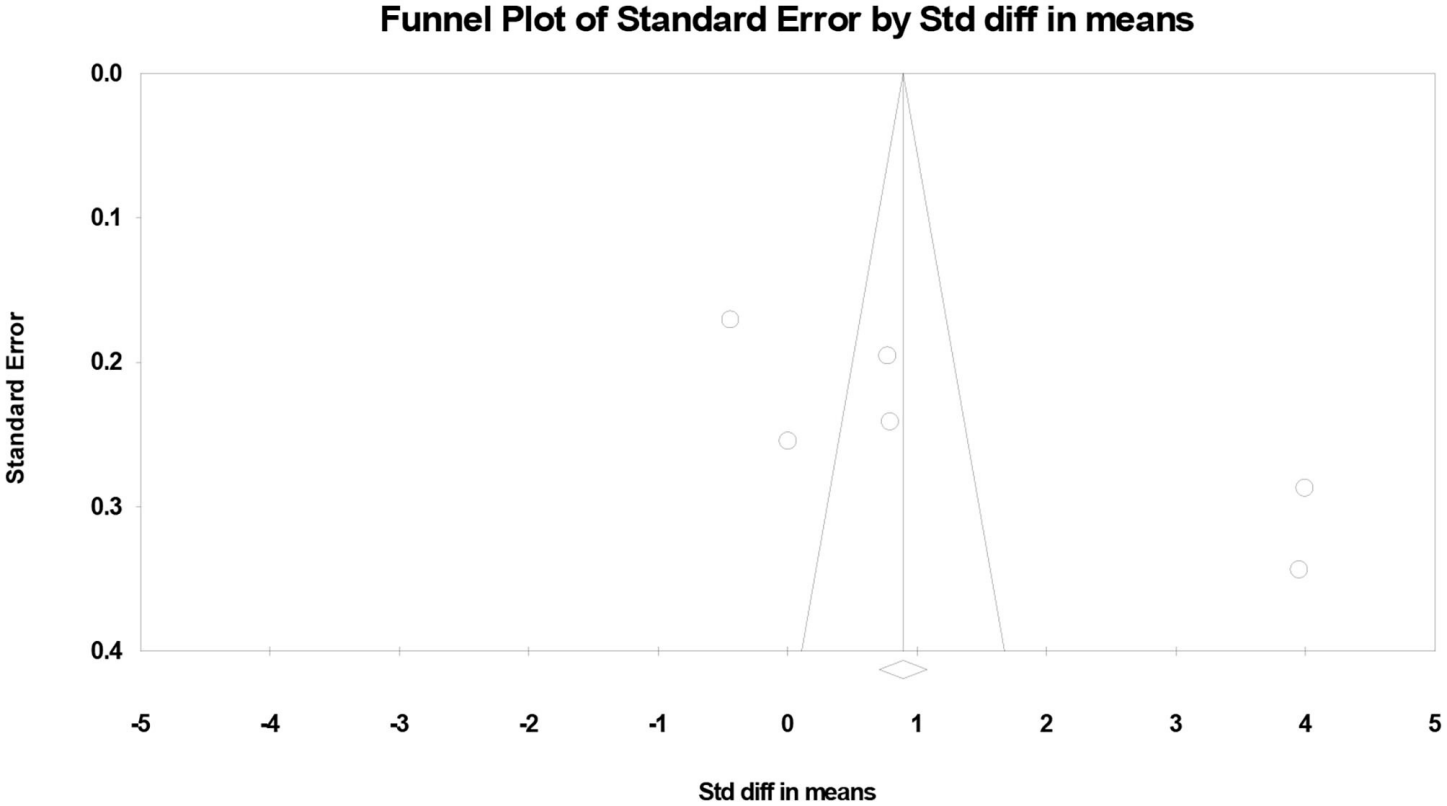
Funnel plots showing the association between vitamin D levels with the risk of urinary tract infection in children.

Finally, we created funnel plots to explore the possibility of publication bias, yet the results of Egger's test were evident of this bias (*p* = 0.041) (Figure 3).

## Discussion

In this meta-analysis we hypothesized that low level of vitamin D acts as a risk factor for UTI in children. Therefore, we included and analyzed six human studies that examined the association between serum vitamin D level and UTI in children. The results of the meta-analysis of these studies showed that there is a negative relationship between vitamin D deficiency and the risk of UTI in children.

A systematic review and meta-analysis by Deng et al. has accumulated the evidence on the association between serum vitamin D level and the risk of UTI (19). They have included 9 studies included 1,921 participants, of which 580 were diagnosed with UTI. Among these studies only four studies had focused on UTI in pediatric population. Two more studies on this population are included in our meta-analysis. Like our results they showed significant association between low levels of serum vitamin D and the risk of UTI in both adult and children. Similar to the current meta-analysis they reported moderate heterogeneity in the included studies. However, our results including six studies the Egger's test was in favor of publication bias in the included studies which was not reported in the Deng et al. study when the results of all adult and children population were included in the test.

Shalaby et al. (15) defined serum 25(OH)D3 level of ≤25 nmol/L as vitamin D deficiency. Data from the study by they showed that serum vitamin D levels in children with UTI (0.98 ± 1.15 years) were 10.5 ± 2.7 nmol/L and in healthy children (0.90 ± 1.23 years) were 27.9 ± 5.6 nmol/L (15). They have concluded that there is a risk of vitamin D deficiency in the pediatric population in the age range studied by these researchers. Moreover, Karatekin et al. (20) reported that serum 25(OH)D levels in children under 2 years are highly correlated with maternal serum levels of 25(OH)D. In addition, we know that maintaining a sufficient serum 25(OH)D level is achieved by consuming a vitamin D rich diet and exposure to sunlight, especially UVB rays for 15–30 min a day. So, consideration of nutrition of the mother and vitamin D levels for both mother and infant are important confounding factor in these studies. These results indicated that newborns and mothers can be susceptible to vitamin D deficiency and infectious diseases (21). They have suggested vitamin D supplementation during pregnancy, lactation and early childhood as a preventive measure for childhood infections including UTI.

Although we did not examine the association between age and sex with plasma levels of vitamin D and the risk of UTI in this study, most studies suggest that plasma vitamin D levels vary with age and sex and it could be one of the limitations of this study. Shalaby et al. (15), Tekin et al. (14), and Georgieva et al. (16) showed that serum 25(OH)D3 levels in girls were significantly lower than boys. This difference in this period of life can be due to malnutrition, inadequate exposure to sunlight and the presence of differencein sex chromosomes (22). Moreover, Georgieva et al. (16) showed that the levels of 25(OH)D3 negatively correlated with age, however, Shalaby et al. (15) reported that there was no correlation between serum 25(OH)D3 levels and age in case and control groups. However, Mahyar et al. (11) and Övünç Hacihamdioglu et al. (18) indicated that there was no significant difference between children with UTI and control in terms of sex, age, weight, height, head circumference and duration of breastfeeding.

Overall, the main finding of this meta-analysis is that there is a negative correlation between low level of serum vitamin D and risk of UTI. Now a question arises that what is the role of vitamin D in the pathogenesis of urinary tract infection?

Vitamin D is a steroid hormone which is mainly produced in the skin after exposure to ultraviolet radiation. The active form of vitamin D is 1, 25(OH) 2 D, that its biological activity appears to be dependent on the plasma concentration of 25(OH)D. According to the European Society for Pediatric Gastroenterology, Hepatology and Nutrition (ESPGHAN) vitamin D deficiency is defined as having a serum/plasma level of <25 nmol/L (10 ng/mL), insufficient between 25 and 50 nmol/L (10–20 ng/mL), adequate between 50 and 75 nmol/L (20–30 ng/mL) and optimal for a level above 75 nmol/L (30 ng/mL) (23).

In addition to the popular role of vitamin D in bone and calcium homeostasis. It can also regulate the innate and adaptive immune function through various mechanisms (21, 24, 25). In fact, the missing link between vitamin D deficiency and the risk of UTI in children appears to be the role of vitamin D in innate and adaptive defense. In children and infants with poorly developed immune systems, vitamin D deficiency can limit the body's ability to deal with various infectious agents.

Vitamin D directly or indirectly regulates the function of innate and adaptive immunity through various mechanisms. It can regulate the inflammatory response to the pathogenic agents (21, 24). Adequate level of vitamin D reduces local and systemic inflammatory responses by modulation of cytokine responses and reduction of toll-like receptor activation in immune cells (26). Vitamin D deficiency not only reduces immune cells functions through the induced hypocalcemia, but also reduces the production of cathelicidin and b-defensin-2 (27). Immune cells such as T-cell, dendritic cells, macrophages and monocytes have vitamin D receptors which regulate their antimicrobial function (28, 29).

Vitamin D receptor is a nuclear receptor that mediates the function of 1,25-dihydroxyvitamin D, the active form of vitamin D. Vitamin D receptor signaling also regulates structural integrity and transport functions of different epithelial barriers (30). It is shown by Aslan et al. (29) that vitamin D receptor gene polymorphism is involved in the pathogenesis of UTI. Endogenous antimicrobial peptides (AMPs) are highly expressed in barrier sites especially urinary tract epithelium, and thus provide a first-line defense mechanism for the innate immune system to respond to infectious agents. They are multi-functional peptides at epithelial surfaces including the skin, gastrointestinal, respiratory and urinary tracts. AMPs show antibacterial activity by destroying the bacterial membrane and inhibit biofilm formation and modulate various immune processes (31). On the other hand, it was reported that AMPs activity in the epithelial surfaces is associated with UTI severity (32).

It should be noted that vitamin D deficiency plays a significant role not only in preventing the infection of urinary tract but also in many other diseases such as pneumonia, chronic fatigue syndrome, myocardial infarction, rheumatoid arthritis, psychosis, osteomalacia in adults and rickets in children (21, 33–37).

There are important limitations affecting the interpretation of our results. Factors such as non-uniform diagnostic criteria in studies, non-uniform pathogenesis in this disease, differences in climate and exposure to sunlight, lack of nutritional status of children with UTI, history of recurrence, and lack of a comprehensive definition of vitamin D deficiency and polymorphisms of vitamin D receptors are the some confounding factors that have been addressed in very few studies. Environmental and dietetic factors also significantly affect the serum vitamin D level and should be controlled in the well-designed studies.

The limited number of studies on the association of the serum vitamin D and risk of UTI enfaced the meta-analysis to great potentials of bias and heterogeneity. We have performed two statistical strategies to manage the mentioned potentials of bias and heterogeneity. Funnel plots was created and Egger's test was performed to explore the possibility of publication bias, yet the results were evident of this bias. Subgroup analysis based on study design was also performed. This analysis showed that only in cross-sectional studies there was a significant difference between vitamin D level in UTI and failed to find any significant association in case-control and prospective cohort studies. Beside the mentioned confounders in the enrolled studies, previous evidence showed that calculating outcome estimates based on the fully adjusted models will not significantly alter the final result of the meta-analysis (19), and also the limited number of available studies we did not applied confounders in the final analysis.

The result of a systematic review and meta-analysis suggest that low serum levels of vitamin D are associated with the risk of UTI in children. However, due to the mentioned heterogeneity and the effect of confounding factors, cautious interpretation of the result should be made and more studies are needed to make conclusive statement on the observed effect.

## Author Contributions

YL designed and supervised the review and wrote the first draft of manuscript. XL, QY, and FQ reviewed the literature and extracted the data. BZ analyzed the data. All authors critically revised and approved the final version of the manuscript.

## Conflict of Interest

The authors declare that the research was conducted in the absence of any commercial or financial relationships that could be construed as a potential conflict of interest.
